# Affecting of Glyphosate Tolerance and Metabolite Content in Transgenic *Arabidopsis thaliana* Overexpressing *EPSPS* Gene from *Eleusine indica*

**DOI:** 10.3390/plants14010078

**Published:** 2024-12-30

**Authors:** Jingchao Chen, Zhiling Li, Haiyan Yu, Hailan Cui, Xiangju Li

**Affiliations:** State Key Laboratory for Biology of Plant Diseases and Insect Pests, Institute of Plant Protection, Chinese Academy of Agricultural Sciences, Beijing 100193, China; chenjingchao@caas.cn (J.C.); yuhaiyan@caas.cn (H.Y.); cuihailan@caas.cn (H.C.)

**Keywords:** *Arabidopsis thaliana*, glyphosate, transgenic plant, secondary metabolites, targeted metabolome

## Abstract

Long-term use of the global non-selective herbicide glyphosate for weed control has caused resistance in weeds. Overproducing of the target of glyphosate 5-enolpyruvylshikimate-3-phosphate synthase (EPSPS) is one of the resistance mechanisms in weeds. However, few studies have measured the effects on tolerance levels and metabolite content in model plant species overexpressing *EPSPS* from weeds. We assessed the resistance levels of transgenic *Arabidopsis thaliana* overexpressing *EPSPS* from *Eleusine indica*, and its effects on metabolite content using the method of both quasi-targeted and targeted metabolomics. The results showed that the average resistance index of the transgenic lines was 4.7 and the exogenous *E. indica EPSPS* expression levels were 265.3- to 532.0-fold higher than those in the wild-type (WT) line. The EPSPS protein ranged from 148.5 to 286.2 μg g^−1^, which was substantially higher than that in the WT line (9.1 μg g^−1^). 103 metabolites associated with flavone and flavonol biosynthesis, the metabolism of aromatic amino acids, energy metabolism, and auxin synthesis were significantly higher in the transgenic glyphosate-resistant individuals (R) than in the WT individuals. The results of quantitative analysis show that pyruvate, sedoheptulose 7-phosphate, and gluconic acid amounts in R plants were 1.1-, 1.6- and 1.3-fold higher than those in WT plants, respectively. However, both citric and glyceric acid levels were 0.9-fold lower than those in WT plants. The abundance of other metabolites in the glycolytic and pentose phosphate pathways of central carbon metabolism was similar in the WT and transgenic plants. Glutamic acid was significantly more abundant in the transgenic line than in the WT plants. In contrast, asparagine, glutamine, and lysine were less abundant. However, the concentration of other amino acids did not change significantly. Overexpression of *E. indica EPSPS* in *A. thaliana* conferred a moderate level of tolerance to glyphosate. Metabolites associated with flavone and flavonol biosynthesis, the metabolism of aromatic amino acids, and energy metabolism were significantly increased. The results of this study will be useful for evaluating the characterisation and risk assessment of transgenic plants, including identification of unintended effects of the respective transgenic modifications.

## 1. Introduction

Weeds are a class of plants that interfere with crop growth, reduce crop yield, and pollute agricultural products [1]. Chemical weed management is considered an efficient way to control weeds because it has a better weeding effect, reduces labor, and ensures food security [2]. The widespread use of genetically modified herbicide-tolerant crops has rapidly increased the use of target herbicides, including glyphosate (*N*-phosphonomethylglycine), one of the most important herbicides worldwide [3]. Glyphosate tolerance is a main trait in existing genetically engineered (GE) crops, accounting for nearly 80% of the GE crop cultivation area [4]. This herbicide effectively inhibits 5-enolpyruvyl-shikimate-3-phosphate synthase (EPSPS EC 2.5.1.19) by binding to it to form a structurally stable EPSPS-S3P-glyphosate complex, which accumulates shikimic acid and blocks aromatic amino acid biosynthesis [5].

Widespread glyphosate use has resulted in the evolution of glyphosate-resistant (GR) weeds worldwide [6]. The mechanisms of glyphosate resistance in weeds can be classified into target sites (TSR) and non-target sites (NTSR) [7]. *EPSPS* overexpression is a TSR mechanism commonly caused by *EPSPS* amplification on chromosomes [8]. It can provide sufficient target enzymes to combine with glyphosate and maintain normal plant growth. The first case of GR *Amaranthus palmeri*, endowed with *EPSPS* amplification, was reported in 2010, and several other GR species have been subsequently reported, such as *Bromus diandrus*, *Lolium perenne* ssp. *multiflorum*, and *Kochia scoparia* [6,9,10]. Similar to this GR mechanism, glyphosate-tolerant GE crops have been obtained by increasing the EPSPS enzyme content, commonly by fusing a modified exogenous *EPSPS* with a strong promoter [11]. GE glyphosate-tolerant crops not only have high-level glyphosate tolerance, but also show fitness traits such as fertility, metabolite content, and hormone biosynthesis [12].

*Eleusine indica*, one of the worst weed species, is a C_4_ annual weed infesting many crops worldwide, including tea, corn, soybean, cotton, rice, and sugarcane, as well as in orchards [13]. Studies on the resistance mechanisms in GR *E. indica* have focused on single or double amino acid substitutions in EPSPS, amplification, and *EPSPS* mutations plus overexpression [14,15,16]. Although *EPSPS* overexpression has been confirmed to be a resistance mechanism in GR *E. indica*, few studies have focused on the function of *EPSPS* gene from *E. indica* in the model plant *Arabidopsis thaliana*. This study aimed to clarify the glyphosate resistance levels of in transgenic *A. thaliana* overexpressing *EPSPS* from *E. indica* and verify whether exogenous *EPSPS* overexpression changes metabolite content, including central carbon metabolites and amino acids, in transgenic *A. thaliana* plants.

## 2. Results

### 2.1. Glyphosate Tolerance in Transgenic Arabidopsis

**Figure 1:**
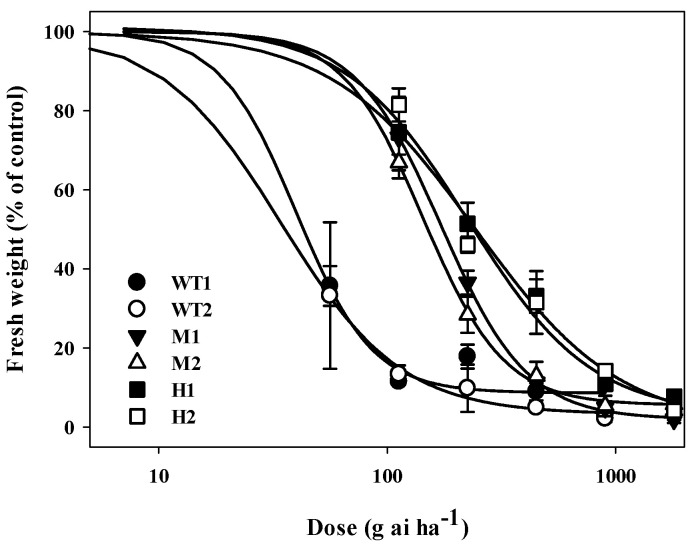
Dose–response curve of transgenic *Arabidopsis thaliana* overexpressing *EPSPS* from *Eleusine indica* to glyphosate 14 days after treatment. Two lines with a relatively high level of *EPSPS* expression named “H1”, “H2”; two lines with medium level *EPSPS* expression named “M1”, “M2”; two WT lines named “WT1”, “WT2”.

In two *A. thaliana* transgenic lines with a relatively high level of *EPSPS* expression named “H1” and “H2”, two lines with medium-level *EPSPS* expression named “M1” and “M2”, and two WT lines named “WT1” and “WT2”, tolerance levels to glyphosate were detected. The results of the whole-plant assay showed that transgenic plants could grow well under 225.0 g ai ha^−1^ glyphosate. However, WT plants were in a state of death with a dose of 112.5 g ai ha^−1^ (Figure 1). Compared with WT plants (GR_50_ = 37.1), the average resistance index (RI) of the transgenic lines with *EPSPS* gene overexpression at the medium (M1, M2) and high levels (H1 and H2) were 4.6 and 6.9, respectively (Table 1). The seeds of these lines were cultured in 1/2 MS medium containing glyphosate (0.1, 0.5 mM; Figure S1). The transgenic lines could grow with roots and green leaves in media containing 0.1 mM glyphosate, whereas the WT lines were yellow and did not grow. The transgenic lines appeared dead and had few roots in the medium containing 0.5 mM glyphosate.

### 2.2. EPSPS Expression and Protein Content Analysis

**Figure 2:**
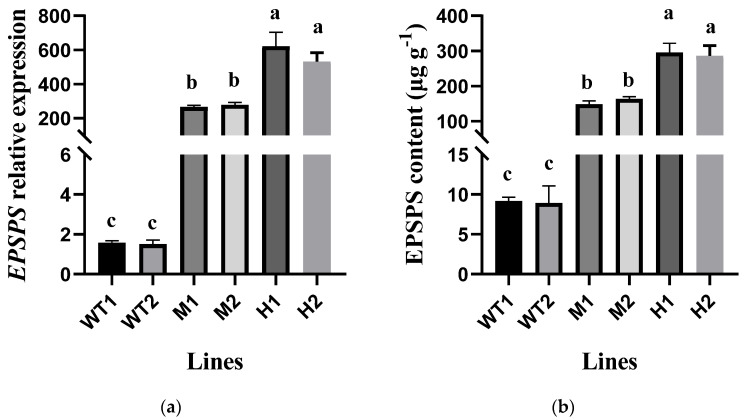
The exogenous *EPSPS* expression levels in transgenic *Arabidopsis thaliana* lines (**a**) and EPSPS protein content (**b**) in different *A. thaliana* lines. The different lowercase letters indicate significant differences in the parameters between the populations. Two lines with a relatively high level of *EPSPS* expression named “H1”, “H2”; two lines with medium level *EPSPS* expression named “M1”, “M2”; two WT lines named “WT1”, “WT2”.

Without glyphosate treatment, exogenous *E. indica EPSPS* expression in the four transgenic *A. thaliana* lines (M1, M2, H1, and H2) was significantly higher than the reference gene. It was approximately 265.3- and 278.0-fold higher in the M1 and M2 lines, respectively, and 621.1- and 532.0-fold higher in the H1 and H2 lines, respectively, than the internal reference gene expression. However, it was almost undetectable in the WT1 and WT2 plants (Figure 2). The EPSPS protein content in M1 and M2 lines were 148.5 and 163.9 μg g^−1^, respectively. The EPSPS protein content in H1 and H2 lines were 295.9 and 286.2 μg g^−1^, which was obviously higher than that in the two WT lines (9.2 and 8.9 μg g^−1^, respectively).

### 2.3. Differences in Metabolite Content

**Figure 3:**
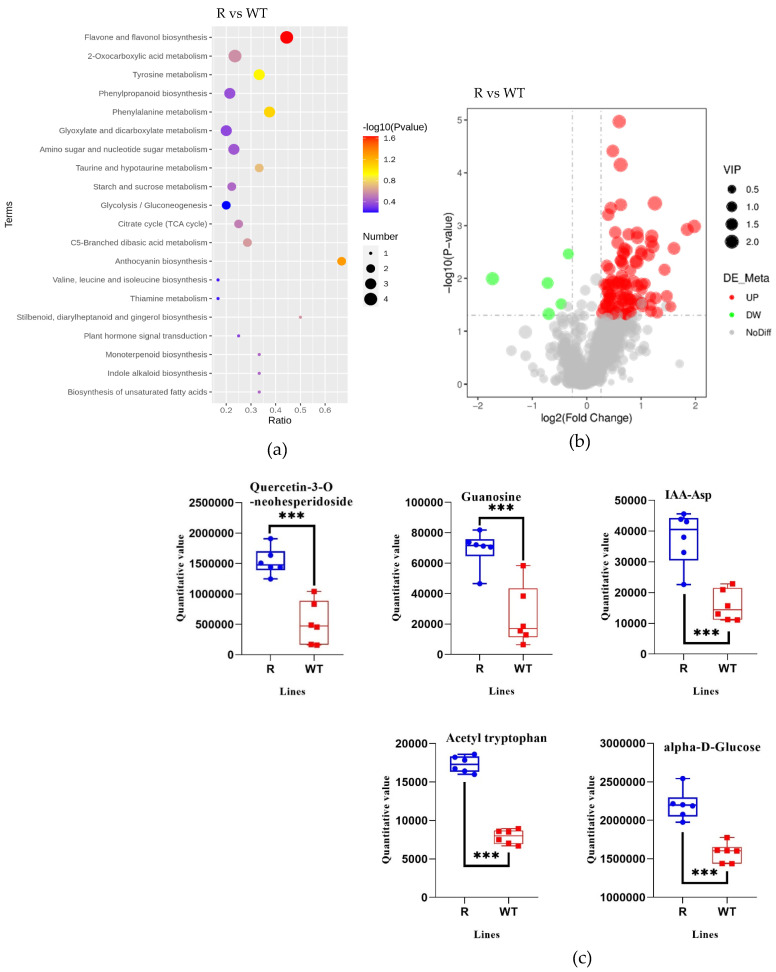
Quantification of the different contents of metabolites in transgenic Arabidopsis thaliana line (R) and wild-type (WT) plants (n = 6) using quasi-targeted metabolomics analysis. (**a**) KEGG enrichment bubble diagrams. (**b**) Volcanic map of differential metabolites. (**c**) Metabolites showing different content in different pathways. Significant differences indicated by “***” (*p* < 0.001) by Student’s *t*-test.

Based on the different database results and screening criteria (VIP > 1 and *p* < 0.05), 108 differential metabolites of the total detected 905 metabolites were selected for comparison between a transgenic line (R) and WT individuals (Figure 3). Among these metabolites, 103 were upregulated, while only 5 were downregulated in R plants relative to those in WT plants (Figure 3). Analysis results show that metabolites associated with flavone and flavonol biosynthesis, the metabolism of Tyrosine (Tyr) and Phenylpropanoid, and some pathways associated with energy metabolism and auxin synthesis were significantly higher in the R individuals than in the WT individuals (Figure 3). For example, the content of quercetin-3-O-neohesperidoside, guanosine, IAA-Asp, acetyl tryptophan, and alpha-D-Glucose in the R individuals was 2.9, 2.8, 2.4, 2.2, and 1.4 times higher than that in the WT individuals, respectively (Figure 3).

### 2.4. Content of Central Carbon Metabolism and Amino Acids

**Figure 4:**
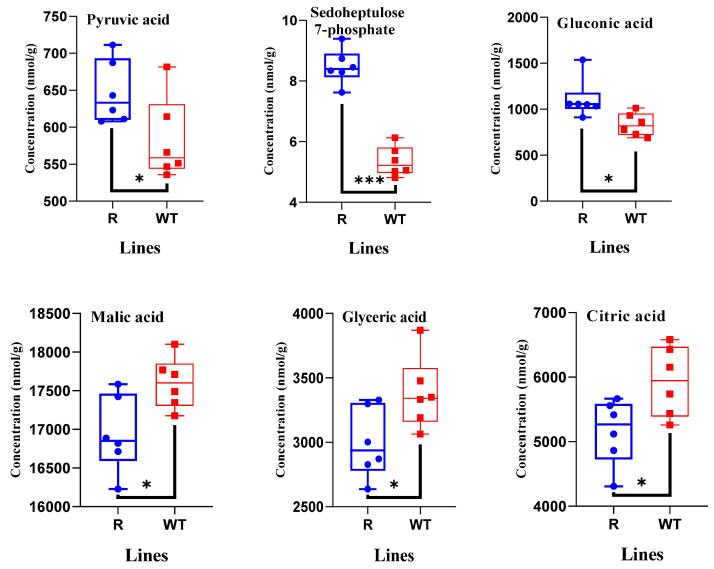
Quantification of the metabolites relevant to target central carbon metabolism (n = 6) in transgenic *Arabidopsis thaliana* line (R) and wild-type (WT) plants. Significant differences indicated by “*” (*p <* 0.05), and “***” (*p <* 0.001) by Student’s *t*-test.

**Figure 5:**
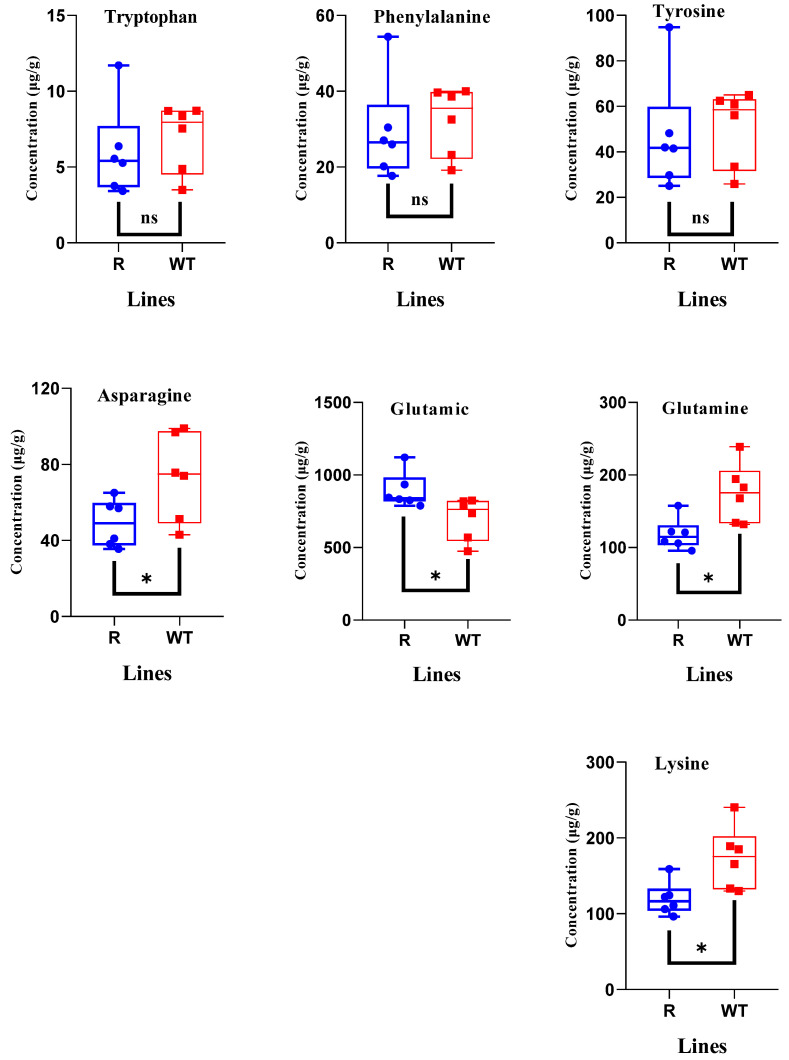
Quantification of different amino acids in the plants of transgenic *Arabidopsis thaliana* (R) and wild-type (WT) plants (n = 6). Significant differences indicated by “*” (*p* < 0.05) by Student’s *t*-test, “ns” mean difference not significant.

The pyruvate, sedoheptulose 7-phosphate, and gluconic acid amounts in R plants were 1.1-, 1.6- and 1.3-fold higher than those in WT plants, respectively (Figure 4). However, the citric, malic, and glyceric acid levels in the R plants were only 0.9-fold lower than those in the WT plants, respectively (Figure 4). The abundances of fructose 6-phosphate, phosphoenolpyruvic acid, and 3-phosphoglyceric acid in the glycolytic pathway and concentration of ribose 5-phosphate in the pentose phosphate pathway in R plants were similar to those in WT plants (Table 2). Similar trends were observed for ATP and ADP levels (Table 2). Twenty-one amino acids were detected, and glutamic acid was significantly more abundant in R plants than in WT plants (Figure 5). In contrast, the amino acids asparagine, glutamine, and lysine showed lower abundance in R than in WT plants (Figure 5). Other amino acids, including the three aromatic amino acids, did not show significant changes in concentration (Table 3, Figure 5).

## 3. Discussion

In studies on gene function verification for herbicide-resistant weed species, resistant genes are usually transferred to model plants, such as *A. thaliana,* by transgenic technology, and the resistance function of the detected genes is assessed by the symptoms of transgenic plants or other parameters after treatment with the target herbicides [17,18,19]. However, owing to the long operation cycle, most researchers use microorganisms such as *Escherichia coli* for glyphosate resistance verification [15,20,21]. In this study, we found that overexpression of the WT *EPSPS* gene from *E. indica* in *A. thaliana* resulted in moderate resistance to glyphosate, with RIs ranging from 4.2 to 7.1. This result was similar to that of a previous study in which *EPSPS* overexpression (5.4 to 29.7 times higher than the susceptible population) in *E. indica* populations conferred glyphosate resistance with RIs from 4.9 to 9.4 [22]. The seeds seemed more sensitive to glyphosate, and root elongation in all transgenic *A. thaliana* lines was significantly inhibited after treatment with 0.1 mM. In contrast to the WT *EPSPS* gene without any mutations, many studies have focused on the glyphosate resistance levels of mutant EPSPS in transgenic *A. thaliana* or *Oryza sativa*. *A. thaliana* plants overexpressing the *E. indica EPSPS* gene with a double mutation TIPS (The102Ile + Pro106Ser) can survive after treatment with 3360 g ae ha^−1^, which is more tolerant to glyphosate than that without mutation in this study [23]. Similar results were also found in transgenic *O. sativa*; the individuals overexpressing *E. indica EPSPS* with the TIPS mutation also displayed higher glyphosate resistance levels, surviving after treatment with 11,255.6 g ae ha^−1^ [24]. In another transgenic *O. sativa*, overexpression of endogenous *EPSPS* genes with a double mutation (T173I + P177S) resulted in survival in half-strength MS media with a glyphosate dosage of 100 mM; however, the WT plants died after treatment with 0.1 mM [12]. All reported results and findings in this study indicate that overexpression of both endogenous and exogenous *EPSPS* genes with or without the mutation in position 102 or 106 increased glyphosate tolerance.

*EPSPS*-overexpressing individuals produce large amounts of EPSPS protein [25]. When treated with glyphosate, parts of the EPSPS protein ensure normal catalytic function and the surplus EPSPS proteins bind to glyphosate to reduce its effect. Several studies have focused on the effect of the abundant EPSPS protein in plants without glyphosate treatment, and some studies found that transgenic *O. sativa* overexpressing the mutant endogenous *EPSPS* genes produced 17–19% more seed weight with higher phenylalanine and tryptophan contents than the WT [12]. A similar result was found in a transgenic F_2_ rice–weedy rice hybrid that overexpressed the modified native *EPSPS* gene. This line produced 48–125% more seeds per plant and had greater EPSPS protein levels, tryptophan concentrations, photosynthetic rates, and percent seed germination than the WT plants [26]. A transgenic *A. thaliana* overexpressing the native *EPSPS* gene driven by the CaMV35S promoter produced 23–37% more seeds per plant than the WT line [27]. We found similar results in a *E. indica* population that evolved glyphosate resistance caused by *EPSPS* overexpression; seed production in this resistant population was 1.4 times higher than that in the WT population. In this study, regarding secondary metabolites relative to flavone and flavonol biosynthesis, the metabolites of metabolism of aromatic amino acids were detected to be increased in R plants by quasi-targeted metabolomics analysis. In addition, the pyruvate and sedoheptulose 7-phosphate levels, which are related to carbon and energy metabolism, increased content in the transgenic lines. This indicated that an adequate level of EPSPS protein caused by high-level *EPSPS* gene expression up-regulates the metabolites in the shikimic acid pathway.

Multi-omics methods were selected to analyse food safety hazards, as well as to identify their composition and origin [28]. The technology of untargeted metabolomics analysis was used or considered using to evaluate the risk of foods and feeds from genetically modified (GM) crops under the appropriate guidance by the European Food Safety Authority (EFSA) [29,30,31,32]. The unexpected fitness traits of the GE crops, such as increased fecundity, altered seed germination, and altered metabolic content, may be due to the positional effect of gene insertion or possible linkage with neighboring sequences [33]. Consistent with this hypothesis, *EPSPS* was overexpressed in GR *E. indica* by the amplification of this gene, and other genes were also co-amplified in telomeres [8]. Similarly, in this research, differences in *EPSPS* gene expression may be relative to the positional effect of gene insertion. The reproductive trait of transgenic *EPSPS* plants and its effect on the metabolite content in tissues may be related to photosynthesis, nutrient content in the culture substrate, and other unexpected factors, which makes the issue very complex and requires extensive research.

## 4. Materials and Methods

### 4.1. Plant Material

An *A. thaliana* strain, Columbia (coded as wild-type; WT), was used as the parent to produce comparative transgenic lineages. The vector pCambia1301 was selected to overexpress the *EPSPS* genes driven by the promoter pCaMV35S, and *EPSPS* was cloned from a glyphosate-susceptible *E. indica* plant. Neomycin phosphotransferase (nptII) was selected as a marker gene to confer kanamycin tolerance. Transgenic *A. thaliana* was obtained by the standard method of *Agrobacterium tumefaciens* infection when the plants reached the flowering stage [34]. All genetically transformed plant seeds were germinated on 1/2 MS culture media containing 10 g/L sucrose, 2.2 g/L M519 (PhytoTech Labs, Lenexa, KS, USA), 3 g/L gelrite gellan gum, and 50 ng/L kanamycin (pH 5.74). All surviving plants (T_0_) with two sets of true leaves on the culture media were transplanted into the soil to obtain T_1_ generation seeds, and overexpressing T_1_ progenies were selected [35]. To obtain homozygous *Arabidopsis* plants, the positive plants (T_3_) were retained after selection for the T_1_ and T_2_ generations and two lineages with different *EPSPS* expression were selected.

### 4.2. Glyphosate Tolerance Assay of Transgenic Arabidopsis

All *Arabidopsis* seeds were kept at 4 °C for 2 days. After surface sterilization of *Arabidopsis* seeds, WT plants and T_3_ homozygous seeds were laid on 1/2 MS media with 0, 0.1, or 0.5 mM glyphosate potassium salt (Zhejiang Wynca Chemical Group Co., Ltd., Jiande, China). The medium formulation was prepared as described above, without the addition of kanamycin. The Petri dish containing the media was maintained under the conditions described above for a week. Growth state and root length at different doses were investigated to evaluate glyphosate tolerance [36].

All *Arabidopsis* lines and transgene-absent plants were cultured in pots (8 × 8 cm^2^) and kept in a plant incubator (Conviron, Winnipeg, MB, Canada) under conditions of (25/23 °C) day/night temperature with 50% relative humidity under 12 h light and 12 h darkness (200 μmol^−2^ s^−1^) [37]. The plants were sprayed with 41% glyphosate isopropylamine salt (Roundup, Monsanto, St. Louis, MO, USA) using a moving cabinet sprayer (TeeJet^®^ XR8002) 21 days after transplanting. Briefly, 0, 112.5, 225, 450, 900, or 1800 g ae ha^−1^ glyphosate was applied to transgenic *Arabidopsis* and 0, 56.25, 112.5, 225, 450, and 900 g ae ha^−1^ glyphosate was applied to transgene-absent *Arabidopsis*. Four replicate pots were sprayed with the same dose. Fresh weight data were collected 14 d after treatment [16].

### 4.3. EPSPS Transgene Expression

Young leaf tissue from lines without glyphosate treatment was collected and stored at −80 °C. Total RNA was extracted using the TRNzol Universal Reagent Kit (Tiangen Biotech Beijing Co., Ltd., Beijing, China) [38]. First-strand complementary DNA (cDNA) synthesis was conducted using the FastKing RT Kit (With gDNase) (TransGen Biotech, Beijing, China) and 1000 ng RNA per sample was selected; the total reaction volume was 20 μL [39]. The *EPSPS* expression levels of transgenic plants were determined using the primers G2F (5′-GGTGGCAAGGTTAAGTTATCTGG-3′) and G2R (5′-TCAACATAAGGGATGGAGATCAG-3′); the reference gene (ACTIN) was amplified using the primers AT-ACT2-F (5′-CTTGCACCAAGCAGCATGAA-3′) and AT-ACT2-R (5′-CCGATCCAGACACTGTACTTCCTT-3′) [15,40]. The expression of *EPSPS* relative to that of the reference gene was expressed as 2^ΔCt^ [41]. The 20 μL RT-qPCR mixture included 1 μL cDNA, 0.5 μL each of the forward and reverse 10 μM primers, 10 μL qPCR SYBR Master Mix, 7.96 μL H_2_O and 0.04 μL ROX (50×). PCR was performed at 50 °C for 2 min; 95 °C for 2 min; 95 °C for 5 s, and 60 °C for 32 s for 40 cycles [25].

### 4.4. EPSPS Protein Content

Fresh leaves were cut from transgenic and WT plants without glyphosate treatment and thoroughly ground in liquid nitrogen. The EPSPS protein content was measured using an enzyme-linked immunosorbent assay (ELISA) kit (Shanghai Youlong Biotech Co. Ltd., Shanghai, China) [42]. Powdered samples (0.1 g) were added to the extract (1.0 mL) and centrifuged at 12,000 rpm for 10 min. Supernatants (0.1 mL) were transferred to antibody-coated plates and incubated for 45 min. Then, 0.1 mL monoclonal multi-antibodies were added to the plate and incubated for 30 min. Chromogenic acid (0.1 mL) was added to the mixture and then incubated for 15 min to determine the solution color. Light absorption at 450 nm was detected using a Tecan Infinite 200 Pro plate reader (Tecan Group Ltd., Männedorf, Switzerland) after adding the stop solution [43]. The EPSPS concentration for each sample was calculated based on the optical density (OD) of the test sample conversion according to a standard curve obtained using purified EPSPS at a known concentration.

### 4.5. Quasi-Targeted Metabolomics Analysis

Tissues (100 mg) of leaves for six transgenic and WT plants without glyphosate treatment were collected. The samples were individually ground with liquid nitrogen, then mixed with prechilled 80% methanol and incubated on ice for 5 min. The supernatant was injected into the liquid chromatography–tandem mass spectrometry (LC-MS/MS) system analysis after centrifugation at 15,000 *g*, 4 °C for 20 min [44]. LC-MS/MS analyses were performed using an ExionLC™ AD system (SCIEX) coupled with a QTRAP^®^ 6500+ mass spectrometer (SCIEX) (Novogene Co., Ltd., Beijing, China). Samples were injected onto an Xselect HSS T3 (2.1 × 150 mm, 2.5 μm) using a 20-min linear gradient at a flow rate of 0.4 mL/min in positive/negative polarity mode [45]. The positive polarity mode was conducted using a QTRAP^®^ 6500+ mass spectrometer with ionSpray voltage of 5500 V at a temperature of 550 °C while the negative polarity was operated using the same method but with an ionSpray voltage was −4500 V.

Detection of the experimental samples using multiple reaction monitoring (MRM) was based on the Novogene in-house database (Novogene, Tianjin, China). The characteristic fragment ion (Q3) was used for metabolite quantification. The specific precursor ion (Q1), Q3, RT (retention time), DP (declustering potential) and CE (collision energy) were used for metabolite identification. The data files generated from high performance liquid chromatography–tandem mass spectrometry (HPLC-MS/MS) were processed using SCIEX OS Version 1.4 to integrate and correct the peaks. The area of each peak represents the relative content of the corresponding substance.

### 4.6. Targeted Metabolomics Analysis

Freeze-dried leaf tissue samples for six transgenic and WT plants without glyphosate treatment were ground into powder at 60 Hz for 30 s. Then, 500 μL precooled MeOH/H_2_O (3/1, *v*/*v*) was added to the powder and weighted samples, and vortexed for 30 s. After homogenizing (4 min at 35 Hz) and sonicating (5 min in an ice water bath) twice, all samples were incubated at −40 °C for 1 h. The supernatant (100 μL) was collected after centrifuging at 13,800× *g* and vacuum-dried. The reconstitution solution was obtained by adding 500 μL ultrapure water to the dried residue, vortex filtered, and transferred to injection vials for analysis. The metabolite standards were mixed and dissolved in ultrapure water (10 mmol L^−1^) as a stock solution, and a series of calibration standard solutions were prepared by stepwise dilution of the stock solution [46]. High-performance ion chromatography (HPIC) was performed using an ICS-6000 HPIC System (Thermo Scientific) equipped with Dionex IonPac AS11-HC (2 × 250 mm^2^) and AG11-HC (2 × 50 mm^2^) columns. The mobile phase A was NaOH (100 mM) and phase B was water. Column and sample temperatures were set to 30 and 4 °C, respectively. An AB SCIEX 6500 QTRAP + triple quadrupole mass spectrometer (MS) (AB Sciex) equipped with an electrospray ionization interface was used for assay development [46]. To improve the accuracy and sensitivity of target metabolite detection, multiple reaction monitoring was employed to analyze the targeted analytes by injecting a standard solution of the individual analyte into the atmospheric pressure ionization (API) source of the MS [46].

### 4.7. Statistical Analyses

Nonlinear regression analysis was selected to analyse the dose-response assay data using the log-logistic equation *Y* = y_0_ + a/[1 + (*X*/*X*_0_)*^b^*], which included four different parameters, and analysis was conducted using SigmaPlot (V12.5; SigmaPlot Software Inc., Chicago, IL, USA) [47]. In this equation, *X*_0_ is the herbicide dose required for 50% plant growth reduction (GR_50_). The resistance index (RI) of the population to glyphosate was calculated as the ratio of the GR_50_ of the resistant lines to that of the WT. Relative *EPSPS* gene expression and protein content were evaluated using analysis of variance with Duncan’s multiple range test at *p* = 0.05, and tissue metabolite quantification data were analyzed using Student’s *t*-test (*p* < 0.05) using SPSS (version 13.0; SPSS, Chicago, IL, USA) [25]. Analyst WorkStation software (1.6.3 AB SCIEX) and MultiQuant 3.0.3. software, and Chromeleon 7 was used for MRM data acquisition and processing. Metabolites with variable importance in the projection (*VIP*) values > 1 and a false discovery rate < 0.05 were considered differentiated [48].

## 5. Conclusions

Overexpression of *E. indica EPSPS* in *A. thaliana* conferred a moderate level of tolerance to glyphosate with an average resistance index of 4.7. Abundant EPSPS resulted in metabolites associated with flavone and flavonol biosynthesis, metabolism of aromatic amino acids, and energy metabolism being significantly increased. The results of this study will be useful for evaluating the physiological impact of overproducing exogenous *EPSPS* in glyphosate-resistant weed and evaluating the characterisation and risk assessment of transgenic plants, including identification of unintended effects of the respective transgenic modifications.

## Figures and Tables

**Table 1:** Parameter estimates (±SE) of regression equations for shoot fresh weight for transgenic *Arabidopsis thaliana* overexpressing the *EPSPS* gene from *Eleusine indica*.

Lines	*b*	*a*	*X*_0_	*R* ^2^	*p*	*RI * ^C^
WT1	2.6 ± 1.0	91.4 ± 3.7	39.9 ± 5.9	0.98	<0.0001	1.2
WT2	1.7 ± 0.8	96.7 ± 6.3	34.3 ± 9.5	0.96	<0.0001	1.0
M1	2.3 ± 0.3	98.0 ± 4.5	172.3 ± 11.6	0.98	<0.0001	5.0
M2	2.4 ± 0.2	94.5 ± 2.1	143.8 ± 4.6	0.99	<0.0001	4.2
H1	1.3 ± 0.1	99.8 ± 9.0	243.9 ± 20.2	0.99	<0.0001	7.1
H2	1.6 ± 0.3	97.9 ± 7.6	227.0 ± 30.1	0.96	<0.0001	6.6

^C^ GR_50_ of transgenic lines divided by GR_50_ of WT line. Parameter *b* is the slope of the curve, *a* is the difference between the upper limit and the lower limit, and *X*_0_ is the GR_50_.

**Table 2:** The central carbon metabolite concentration in the leaf tissue of *Arabidopsis thaliana* overexpressing the *EPSPS* gene from *Eleusine indica* (R) or wild-type (WT) *A. thaliana*.

Metabolites	R (nmol g^−1^)	WT (nmol g^−1^)	*p* Value	Metabolites	R (nmol g^−1^)	WT (nmol g^−1^)	*p* Value
3-Phosphoglyceric acid	6.1	6.7	0.356	Glycolic acid	388.3	386.7	0.972
Adenosine diphosphate	4.7	4.8	0.844	Glyoxylic acid	1044.0	1133.5	0.703
Adenosine triphosphate	2.3	2.4	0.729	Guanosine diphosphate	2.6	2.8	0.337
Fructose 1,6-bisphosphate	10.1	10.4	0.214	Indoleacetic acid	75.8	77.9	0.780
Fructose 6-phosphate	15.5	16.9	0.766	L-Lactic acid	164.9	183.7	0.534
Glucaric acid	195.3	211.2	0.248	Phosphoenolpyruvic acid	5.2	5.3	0.480
Glucose 1-phosphate	5.6	5.5	0.901	Ribose 5-phosphate	1.9	2.0	0.538
Glucose 6-phosphate	106.8	105.1	0.947	Succinic acid	1385.3	1439.5	0.724
Glucuronic acid	23.9	22.7	0.488				

**Table 3:** The amino acid concentration in the leaf tissue of *Arabidopsis thaliana* overexpressing the *EPSPS* gene from *Eleusine indica* (R) or wild-type (WT) *A. thaliana*.

Metabolites	R (µg g^−1^)	WT (µg g^−1^)	*p* Value	Metabolites	R (µg g^−1^)	WT (µg g^−1^)	*p * Value
Glycine	11.8	12.1	0.865	Threonine	68.2	68.5	0.980
Serine	98.5	93.3	0.508	Histidine	21.1	21.5	0.918
Methionine	10.1	11.1	0.754	Valine	10.1	11.0	0.715
Proline	13.7	14.7	0.657	Ornithine	2.5	2.0	0.348
Leucine	36.9	43.4	0.478	Alanine	71.3	71.9	0.956
Aminobutyric	117.9	150.8	0.083	Isoleucine	21.3	25.5	0.523
Argine	117.5	132.7	0.443	Aspartic	384.1	391.1	0.849

## Data Availability

Data are contained within the article and supplementary materials.

## Supplementary Materials

The following supporting information can be downloaded at: https://www.mdpi.com/article/10.3390/plants14010078/s1: Figure S1: The response of *Arabidopsis thaliana* to glyphosate with different expression levels of *EPSPS* gene from *Eleusine indica*. (a) Seed germination in MS medium containing different concentrations of glyphosate. (b) Whole-plant assay to glyphosate.
