# Supplementary material for: Affecting of Glyphosate Tolerance and Metabolite Content in Transgenic Arabidopsis thaliana Overexpressing EPSPS Gene from Eleusine indica
Source: Plants (Basel). 2024 Dec 30;14(1):78. doi: 10.3390/plants14010078 (PMC11723125; doi:10.3390/plants14010078)
Supplement: Supplementary file 1 [file plants-14-00078-s001.zip › plants-3365917-supplementary/plants-3365917-Supplementary files.pdf]

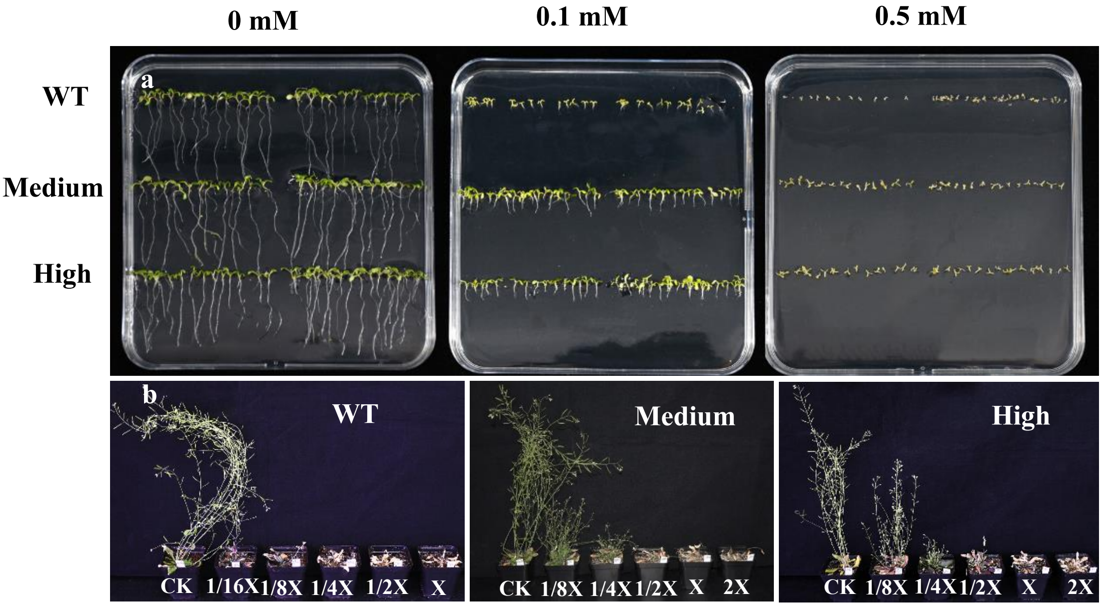

Figure S1. The response of *Arabidopsis thaliana* to glyphosate with different expression level *EPSPS* gene from *Eleusine indica*. (a) Seed germination in MS medium containing different concentrations of glyphosate. (b) Whole-plant assay to glyphosate.
